# Postoperative hepatic encephalopathy following laparoscopic repair of diaphragmatic hernia after radiofrequency ablation: a case report and literature review

**DOI:** 10.1093/jscr/rjaf858

**Published:** 2025-10-26

**Authors:** Junki Fukuda, Akira Shibata, Yuma Hane, Takahiro Saito, Kohei Nishigami, Naoto Senmaru, Satoshi Hirano

**Affiliations:** Department of Surgery, Steel Memorial Muroran Hospital, 1-45, Chiribetsucho, Muroran, Hokkaido 050-0076, Japan; Department of Gastroenterological Surgery II, Hokkaido University Graduate School of Medicine, North 15, West 7, Kita-ku, Sapporo, Hokkaido 060-8648, Japan; Department of Surgery, Steel Memorial Muroran Hospital, 1-45, Chiribetsucho, Muroran, Hokkaido 050-0076, Japan; Department of Surgery, Steel Memorial Muroran Hospital, 1-45, Chiribetsucho, Muroran, Hokkaido 050-0076, Japan; Department of Surgery, Sapporo Higashi Tokusyukai Hospital, North 33, East 14, 3-1, Higashi-ku, Sapporo, Hokkaido 065-0033, Japan; Department of Surgery, Steel Memorial Muroran Hospital, 1-45, Chiribetsucho, Muroran, Hokkaido 050-0076, Japan; Department of Surgery, Steel Memorial Muroran Hospital, 1-45, Chiribetsucho, Muroran, Hokkaido 050-0076, Japan; Department of Gastroenterological Surgery II, Hokkaido University Graduate School of Medicine, North 15, West 7, Kita-ku, Sapporo, Hokkaido 060-8648, Japan

**Keywords:** diaphragmatic hernia, hepatic encephalopathy, radiofrequency ablation, laparoscopic surgery

## Abstract

This case report describes a rare, late complication of radiofrequency ablation for hepatocellular carcinoma. A 77-year-old woman with a history of hepatocellular carcinoma presented with an incarcerated diaphragmatic hernia (DH), which was successfully repaired using emergency laparoscopic surgery. However, 2 months later, she developed hepatic encephalopathy due to severe fecal impaction. Imaging and endoscopy revealed significant colonic stenosis at the hernia repair site. The stenosis was successfully resolved with two sequential balloon dilations, leading to a complete recovery from the encephalopathy. This is the first reported case of secondary hepatic encephalopathy caused by colonic stenosis following laparoscopic repair of DH after radiofrequency ablation. This report demonstrates the feasibility of laparoscopic surgery for DH and emphasizes the necessity for meticulous long-term monitoring to avoid such rare but serious delayed complications.

## Introduction

Radiofrequency ablation (RFA) is an effective and minimally invasive treatment for early-stage hepatocellular carcinoma (HCC). Diaphragmatic hernia (DH), although rare, is a potentially serious late complication following RFA and often necessitates urgent surgical intervention, particularly in cases involving intestinal incarceration [1]. Historically, open surgery has been the standard approach for DH repair. In recent years, however, several recent reports have highlighted the benefits of laparoscopic surgery, which allows both diagnosis and treatment using a minimally invasive approach [2–6].

We report a rare case of successful laparoscopic repair for DH following RFA. Subsequently, the patient developed an uncommon postoperative complication: secondary hepatic encephalopathy due to transverse colonic stenosis at the herniation site. This case underscores the importance of early recognition and minimally invasive management of delayed complications of RFA.

## Case presentation

**Figure 1:**
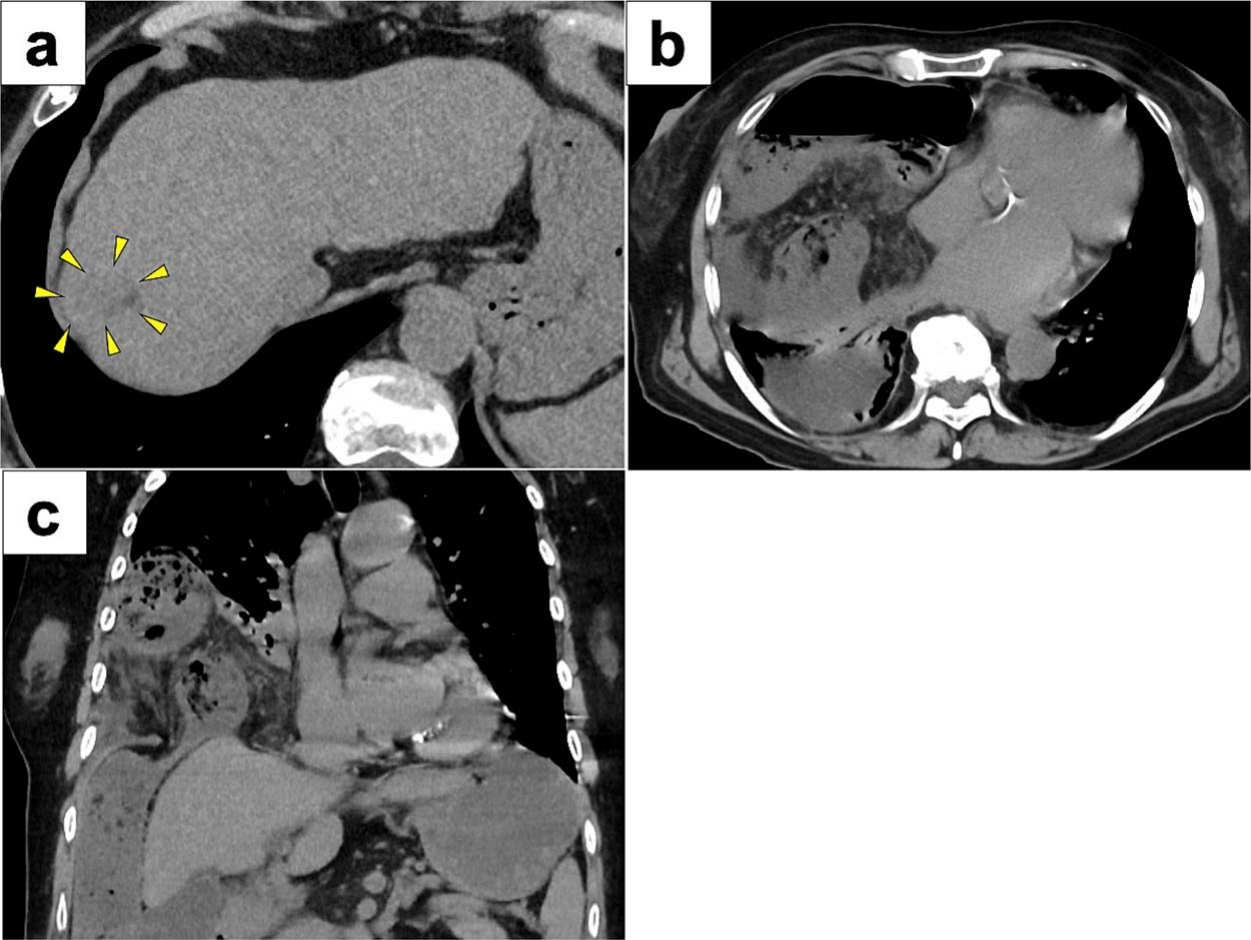
CT images of preoperative examinations. (a) Pre-RFA CT showing that HCC was a 20-mm lesion located in the subcapsular portion of hepatic Segment 8 (arrow). (b and c) Axial and coronal abdominal CT showing incarceration of the transverse colon into the thoracic cavity.

**Figure 2:**
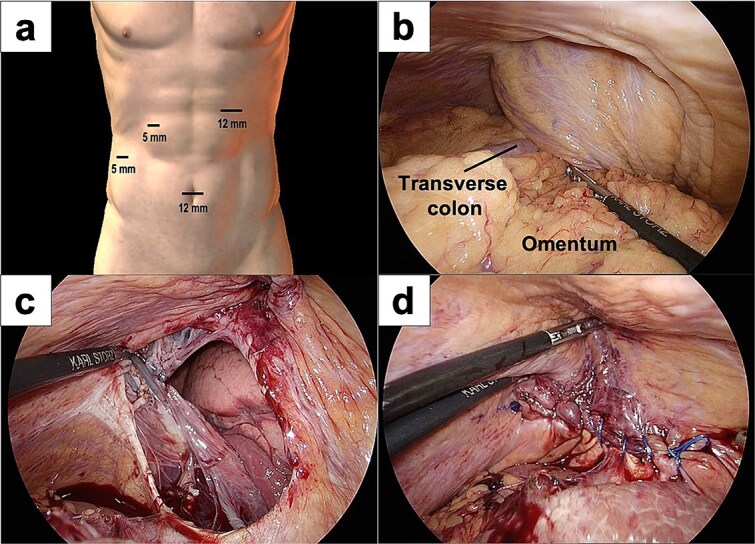
Intraoperative images of laparoscopic surgery. (a) Placement of the laparoscopy trocars. (b) The transverse colon and omentum were found incarcerated in the right diaphragmatic hernia. (c and d) A 50 × 50 mm diaphragmatic defect was identified and closed using barbed sutures.

A 77-year-old women presented to the emergency department with sudden-onset of abdominal pain. Her medical history included alcoholic liver cirrhosis and HCC in liver Segment 8, previously treated with ultrasound-guided RFA 8 months prior. The HCC was a 20-mm lesion located in the subcapsular portion of hepatic Segment 8 (Fig. 1a). Her vital signs were unremarkable. On physical examination, she had marked tenderness in the right upper quadrant. Laboratory tests revealed normal inflammatory markers: white blood cell count of 5330/μl and C-reactive protein 0.1 mg/dl. Regarding hepatic function, she was classified as Child-Pugh score 5, with total bilirubin 1.6 mg/dl, albumin 3.9 g/dl and prothrombin time percentage 77.5%. Computed tomography (CT) imaging demonstrated transverse colon incarceration into the thoracic cavity, with mesenteric edema (Fig. 1b and c). A diagnosis of intestinal incarceration due to DH was made, and emergency surgery was performed. First, a 12-mm trocar was placed at the umbilicus for the scope. Additionally, we placed a 12-mm trocar in the left upper abdomen, a 5-mm trocar in the right upper abdomen, and a 5-mm trocar in the right flank for support (Fig. 2a). Laparoscopic exploration confirmed incarceration of the hepatic flexure (Fig. 2b). The incarcerated colon was carefully reduced, and indocyanine green (ICG) perfusion imaging confirmed adequate blood flow, avoiding the need for intestinal resection. A 50 × 50 mm diaphragmatic defect was identified and repaired using barbed non-absorbable sutures (1-0 V-Loc; Medtronic, Covidien) (Fig. 2c and d). The operative time was 95 min with minimal blood loss. The postoperative course was uneventful, and the patient resumed oral intake on postoperative day 1 and was discharged on postoperative day 8.

**Figure 3:**
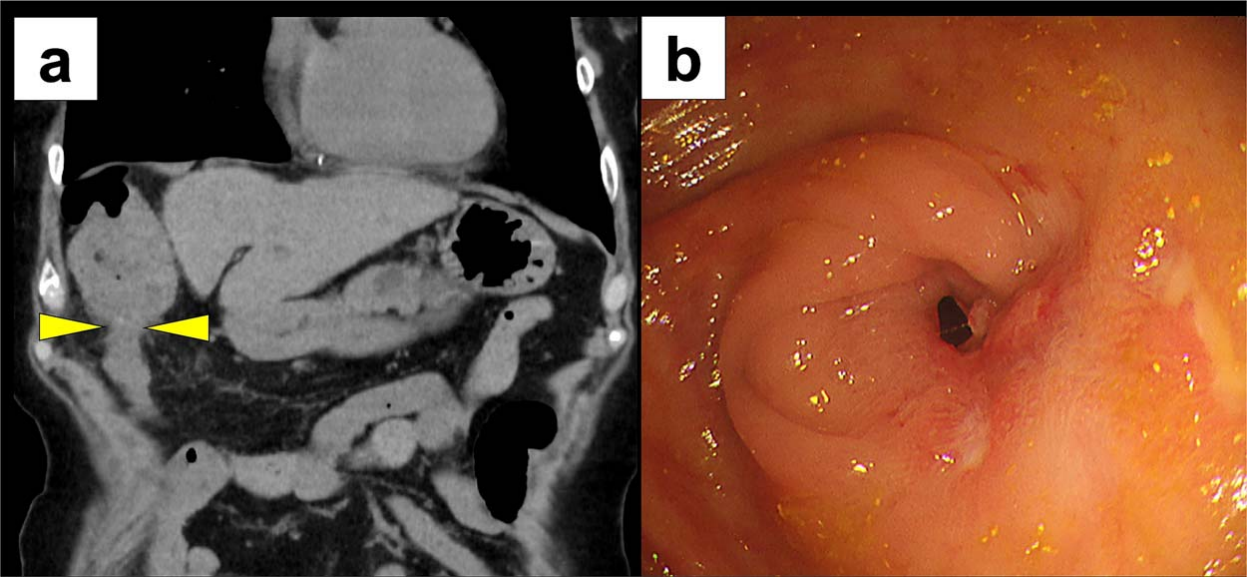
Postoperative imaging studies. (a) CT scan showing stenosis of the transverse colon with massive fecal impaction in the ascending colon (arrow) and massive fecal impaction in the ascending colon. (b) Lower gastrointestinal endoscopy revealing severe stenosis at the hepatic flexure of the transverse colon.

Two months later, the patient was readmitted with disorientation. On arrival, her Japan Coma Scale score was 3. Laboratory findings showed normal liver function (AST 17 U/l, ALT 9 U/l, total bilirubin 1.2 mg/dl) but a markedly elevated ammonia level of 245 μg/dl. CT imaging revealed transverse colon stenosis and massive fecal impaction in the ascending colon (Fig. 3a). Lower gastrointestinal endoscopy showed severe stenosis at the hepatic flexure of the transverse colon, leading to obstruction (Fig. 3b). The stenosis was attributed to ischemic scarring from the previous hernia. A diagnosis of secondary hepatic encephalopathy to constipation due to transverse colonic stenosis was made. Two sessions of endoscopic balloon dilation were performed, resulting in improved colonic patency. We treated the patient with two endoscopic balloon dilations. The first session involved a controlled radial expansion balloon (Boston Scientific) with a diameter of 12 mm. The balloon was inflated to 10 psi for 1 min twice. The second session was performed after 2 weeks and involved the same balloon type, which was inflated to 15 mm. At 8 months follow-up, the patient remains asymptomatic, with no recurrence of DH or hepatic encephalopathy, and continues regular outpatient follow-up.

## Discussion

DH following RFA for HCC is a rare but potentially life-threatening late complication. Mulier *et al*. [1] reported an incidence of DH after RFA was ~0.28% over a 10-year period. Both mechanical and thermal injuries contribute to DH after hepatic RFA. Mechanical injury is caused by the RFA needle, while thermal injury leads to an inflammatory response that weakens the diaphragm, causing a delayed defect [2, 7]. In cirrhotic patients, Chilaiditi syndrome can also increase the risk of DH due to liver atrophy. Clinically, DH typically presents with acute right upper abdominal pain and dyspnea. Contrast-enhanced CT is crucial for diagnosis and for assessing the viability of the incarcerated organs.

While open surgery has traditionally been the primary approach for DH repair, recent reports increasingly highlight the benefits of minimally invasive laparoscopic techniques. Young *et al*. [8] found that laparoscopic repair of Morgagni hernia—a form of DH—was associated with shorter hospital stays compared to open surgery. Surgical treatment for DH involves two approaches: the transabdominal or transthoracic method. In this case, we selected the transabdominal approach because it enabled the safe reduction of the incarcerated bowel and the simultaneous assessment of its blood flow. However, when adhesions are within the thoracic cavity, using a thoracic approach could be beneficial. Table 1 summarizes reported cases of laparoscopic surgery for DH after RFA [2–6]. Among the six patients (2 males and 4 females), the median age was 78 years (range 46–82 years). The time from RFA to DH onset ranged from 8 to 96 months (median 20.5 months), indicating that DH is a delayed complication of RFA. In all cases, the RFA-treated HCC was located adjacent to the diaphragm in the right hepatic lobe, specifically Segment 8. In the reviewed literature, regardless of the hernia orifice size (median 45 mm, range 20–50 mm), suture repair alone was used in four cases, while the remaining two cases involved suture and mesh repair. Several studies of DH have suggested that unidirectional barbed sutures reduce hernia recurrence rates [9], with 2-0 or 1-0 monofilament non-absorbable sutures generally recommended [10]. Furthermore, mesh repair has been shown to be more effective than suture repair alone in reducing infection risk and recurrence rates, particularly for large defects exceeding an average of 73 mm [11, 12]. In our case, mesh repair was avoided due to concerns about potential delayed complications such as intestinal perforation, necrosis, or interference with future RFA. However, based on retrospective data, mesh repair may have been preferable to minimize recurrence risk.

**Table 1:** Summary of laparoscopic surgery for diaphragmatic hernia after radiofrequency ablation.

Author (year)	Age/Sex	Symptoms	Period after RFA (month)	Location of HCC	Hernia orifice (mm)	Surgical procedure	Recurrence of hernia	Postoperative complication
Singh (2011) [2]	46/F	Dyspnea, chest pain	19	S5/8	50	Laparoscopic suture repair	No	No
Nomura (2014) [3]	62/M	Abdominal pain	96	S8	40	Laparoscopic suture repair + mesh	Yes	Hernia recurrence
Ushijima (2021) [4]	82/M	Dyspnea	16	S8	20	Laparoscopic suture repair + mesh	No	No
Kimura (2021) [5]	80/F	Abdominal pain	22	S6/7	20	Laparoscopic suture repair	No	No
Tsunoda (2022) [6]	80/F	Abdominal pain	28	S8	50	Laparoscopic suture repair	No	No
Current case (2025)	77/F	Abdominal pain	8	S8	50	Laparoscopic suture repair	No	Hepatic encephalopathy

M, male; F, female.

Postoperative complications of DH repair can include not only recurrence but also cardiac tamponade, entero-pleural fistula, and adhesive intestinal obstruction [13–15]. To our knowledge, there are no prior reports of secondary hepatic encephalopathy caused by intestinal narrowing at the site of DH repair, as occurred in our case. The frequency of hepatic encephalopathy onset owing to intestinal stenosis remains unclear. However, in patients with pre-existing cirrhosis, it is a rare, potential trigger that may become severe. The pathophysiological mechanisms involve bacterial overgrowth in the small intestine, resulting in the production of ammonia. The intestinal absorption of ammonia leads to elevated blood ammonia levels in patients with liver cirrhosis, as their liver clearance function is impaired. This results in the onset of hepatic encephalopathy. In this case, we used ICG during surgery to evaluate blood flow in the incarcerated intestine; however, intestinal stenosis developed postoperatively. Since ICG fluorescence primarily evaluates blood flow on the serosal surface only, ischemia may have progressed in the mucosal layer, potentially leading to intestinal stenosis. Furthermore, we treated the intestinal stricture in the patient using two endoscopic balloon dilation procedures. Minimally invasive endoscopic dilation was primarily preferred to surgery because, considering the patient's hepatic encephalopathy, major surgery would significantly increase the risk of perioperative complications (such as bleeding, infection, and worsened liver function) and mortality. Additionally, the anticipated complication of reoperation owing to severe adhesions prompted the endoscopic dilation approach. Notably, if endoscopic dilation had improved the intestinal stricture insufficiently, surgical resection would have been performed. In conclusion, we report the first known case of postoperative secondary hepatic encephalopathy following laparoscopic repair of DH after RFA, accompanied by a literature review of similar cases. Once the patient’s respiratory and hemodynamic status is stabilized, emergency laparoscopic surgery for DH after RFA is a feasible and effective treatment option. Additionally, given that RFA patients frequently have underlying cirrhosis, hepatic encephalopathy should be recognized as a potential postoperative complication.
